# Voluntary HIV testing and risky sexual behaviours among health care workers: a survey in rural and urban Burkina Faso

**DOI:** 10.1186/1471-2458-13-540

**Published:** 2013-06-05

**Authors:** Fati Kirakoya-Samadoulougou, Seydou Yaro, Alain Deccache, Paulin Fao, Marie-Christine Defer, Nicolas Meda, Annie Robert, Nicolas Nagot

**Affiliations:** 1 Pôle Epidémiologie et Biostatistique, Institut de Recherche Expérimentale et Clinique (IREC), Faculté de Santé Publique (FSP), Université catholique de Louvain (UCL), Brussels, Belgium; 2 Centre Muraz, Bobo-Dioulasso, Burkina Faso; 3 Institut de recherche santé et société (IRSS), Faculté de Santé Publique (FSP), Université catholique de Louvain (UCL), Brussels, Belgium; 4 Département de santé publique, Université de Ouagadougou, Ouagadougou, Burkina Faso; 5 INSERM U1058 «Infection by HIV and by agents with mucocutaneous tropism: from pathogenesis to prevention, Université Montpellier 1 & CHRU Montpellier, Montpellier, France

**Keywords:** VCT, High-risk sex, Health care workers

## Abstract

**Background:**

Voluntary counselling and testing (**VCT**) together with a safe sexual behaviour is an important preventive strategy in the control of HIV. Although Health care workers (HCWs) are critical in the response to HIV, little is known about VCT and high risk behaviours (HRB) among this group in West Africa. This study aims to assess the prevalence of VCT and HRB among HCWs in Burkina Faso.

**Methods:**

We collected data through a questionnaire in urban areas (Ouagadougou and Bobo-Dioulasso) and rural areas (Poni and Yatenga) among HCWs from 97 health care facilities. Urine samples were collected, screened for HIV using a Calypte® test kit and confirmed by Western Blot. Multiple logistic regression analysis was performed to identify factors associated with the use of VCT services and with high-risk sex behaviour.

**Results:**

About 92.5% of eligible HCWs participated (1570 out of 1697). Overall, 38.2% of them (34.6% of women and 42.6% of men) had ever used VCT services. About 40% of HCWs reported that fear of knowing the test result was the main reason for not doing the HIV test. Male HCWs (p = 0.001), laboratory workers (p < 0.001), those having two years or more experience (p = 0.03), and those who had multiple partners (p = 0.001) were more likely to have tested for HIV. One fifth of HCWs reported multiple partners. Of these, thirteen percent did not use condoms. HCWs who had multiple partners were significantly more likely to be men, single, living in rural areas, and under the age of 29 years.

**Conclusion:**

VCT was still very low among HCWs in Burkina Faso, while HRB was high.

These findings suggest that ‘HCW-friendly’ VCT centres should be implemented, securing confidentiality among colleagues. In addition, refreshment courses on HIV risk reduction, counselling and testing are certainly required during the professional career of HCWs.

## Background

In Sub-Saharan countries, the HIV/AIDS epidemic has eroded more than two-thirds of the Gross Domestic product, by reducing agricultural**-**[1] and companies production [2]. This loss in productivity has been documented in some specific private sectors like mine workers, [2] business workers, [2] construction workers,[3] and transport workers [4]. Key public sectors of development such as Education and Health are also concerned with this negative impact of HIV/AIDS [5]. For public health professionals, the HIV pandemic has increased the demand for health workers, and efforts to scale up HIV prevention, treatment and care rely on the size and capacity of national health workforces. At the same time, the increased global commitment to HIV puts more pressure on bottlenecks created by health workforce shortages, especially in Sub-Saharan Africa where HIV intervention targets remain unmet [6-8]. In light of this, strengthening and expanding the health workforce has been identified as a global challenge to scaling up HIV services [9,10] and the commitment to meeting this challenge has been renewed.

Voluntary HIV testing (VCT) serves as an entry point to HIV/AIDS prevention, care, and support [11]. People unaware of their HIV infection cannot access effective treatment, or at a very late stage, and there is evidence that people who are aware of their HIV status can adopt practices to reduce HIV transmission [12,13]. Knowledge of HIV status remains inadequate: based on 10 population-based surveys conducted in 2007–2009, the median percentage of people living with HIV who know their status was below 40% [14].

In Burkina Faso, VCT in the general population remains very poor. Data from the last demographic and health survey, showed that only 22% of men and 30% of women aged 15–49 have ever been testing for HIV, among them, 20% and 29% knew their HIV status, respectively [15].

In Burkina Faso as in most African countries, HCW should play a great role in informing their patients on HIV and the benefits of HIV testing, particularly when those patients are at risk (multipartnership, STIs, etc. …).. However, there is concern that this information on HIV and by extension on sexual and reproductive health is rarely given to patients. Given HCWs are also at risk through professional and sexual exposure [16,17], their level of VCT utilisation for themselves would be a good indicator of their awareness and ability to address this issue.

Moreover, despite the obvious negative effects of the epidemic on the health sector, no data are available in West Africa on the level of sexual risk for HIV among health care workers, and also on their uptake of HIV testing, which are crucial to inform HIV control programmes. The focus of this study is therefore to assess the proportion of VCT users and the prevalence of high-risk sex (partners other than husband or long-term partner in the last 12 months) among HCWs, together with factors associated with HIV testing.

## Methods

The study was part of a national survey that aimed at estimating the prevalence of HIV among health care workers. Based on an expected prevalence of HIV of 4% and a 95% confidence level, the required sample size was 1476 to get a 1% precision on prevalence estimate. Assuming an acceptability rate for anonymous urinary HIV testing of 65%, the required sample size was 2270 people. Working with 4 provinces, we planned to recruit 600 participants per province, i.e. 2400 overall. Before the study began, the latest statistics on the number of structures, the number of staff and nominal lists by activity and addresses of all HCWs in the selected areas were obtained from the Ministry of Health. A numbering system of survey areas, structures and potential subjects was established. HCWs were selected between January and December 2003 in 97 health care facilities. For rural areas (province of Poni and Province of Yatenga), all health facilities were visited without selection to achieve the sample size. HCWs from urban areas (Ouagadougou and Bobo-Dioulasso: the two biggest towns in the country) were selected by random sampling with probability proportional to size, the size being the number of health agents by health facilities.

About 92.5% of eligible health workers participated (1570 out of 1697); 100% from Province of Yatenga, 96.3% from Ouagadougou, 94.9% from Province of Poni and 84.1% from Bobo-Dioulasso. Ethical clearance was sought and obtained from the Regional Committee for Medical Research Ethics before data collection. The study purpose was discussed with the participants, and after informed consent was obtained, demographic characteristics and informations about HIV testing were collected through an anonymous questionnaire administered by an experienced sociologist. Questionnaires were validated by a 2-month period of field observation and interviews with 30 HCWs in other schools. The questionnaire solicited respondents’ socio-demographic information including health facility, location, sex, age, marital status, HIV testing and sexual behaviour*.* Participants were asked the question, ‘Have you ever been voluntarily tested for HIV to learn your HIV status?’ (Yes or no). This question meant HIV testing in VCT services which always include pre-test and post-test counselling in Burkina Faso. Our dependent variables measured risky sexual behaviour in two ways: i) number of partners, other than husband or long-term partner, in last 12 months (high-risk sex), and ii) the use of a condom during last intercourse with an occasional partner.

Urine samples were obtained from health agents. These samples were screened for HIV using a Calypte test kit and confirmed by Western blot. Twice-reactive samples by Calypte and Western blot were considered HIV-positive. We used an anonymous linked approach whereby the HIV results could not be given back to participants. The latter were encouraged to get a free HIV test and referred to a VCT centre if they wished to know their HIV status. Only ID numbers were used in the questionnaire and matched urine samples.

Chi2 tests, Fisher’s exact tests and Student’s t-tests were used for comparisons at a significance level of 5%. Multiple logistic regression analysis was performed to identify factors associated with the use of VCT services or high-risk sex by checking possible co-linearity and interactions*.* The data were analysed using SAS version 9.3.

## Results

Because of low rate participation among 500 medical doctors (<1%), this report is based on data from non medical doctors.

### Sample characteristics

Among the 1570 HCWs enrolled, 38.2% were from Ouagadougou, 32.0% from Bobo-Dioulasso, 18.9% from Province of Yatenga, and 10.9% from Province of Poni.

The average age of respondents was 34 years (range, 20–60 years), and the majority were women (55.1%). The mean duration of employment was 10 years (range, 1–38 years). HCWs were composed of nurses (30.1%), specialist nurses called “Attaché de santé” (7.8%), auxiliary nurses called “agents itinérant de santé” (6.5%), midwives or auxiliary midwives (21.7%), students and trainees (STU) (15.0%), administrative and manual workers (ADM) (17.5%) and laboratory workers (2.4%). For all analyses, job categories were collapsed into nurses/midwives (NUR/MID) (66.1%), STU (16.4%), laboratory (2.2%), administrative and manual workers (15.2%).

Of respondents, 68.6% were married or living with a regular partner, 23.8% were single and 7.6% were divorced, separated or widowed. Overall, 75.8% of HCWs had a good knowledge of the main routes of HIV transmission (unprotected sexual intercourse, direct injection with HIV-contaminated needles, syringes, blood or blood products, mother to child transmission through pregnancy, delivery or breastfeeding) and more than 42.0% of HCWs had a poor knowledge of the mechanisms of HIV prevention (Abstinence and safe sex, precautions regarding the using of needles, syringes, blood or blood products).

The majority (96.5%) of interviewed HCWs have considered their occupation as being at risk of contracting HIV/AIDS (professional vulnerability for HIV).

The overall prevalence of HIV among HCWs was 3.5% (95%CI: 2.3 – 4.6) with 2.7% (95%CI 1.3 – 4.2) for male HCWs and 4.1% (95%CI 2.4 – 5.8) for female HCWs (p = 0.13).

### HIV testing

Overall, 38.2% (n = 1570) of individuals (34.6% of women and 42.6% of men, p = 0.001) had ever used voluntary counselling and testing services (VCT). The majority of HCWs had tested for HIV at private laboratories (63.0%), at a government health facility (22.5%), and public laboratories (14.5%). Fear of knowing the outcome was the main reason given for not taking the HIV test, as reported by 40.0% of HCWs. More HCWs working in laboratories had reported more HIV testing than other HCWs (p = 0.001) (Table 1). Among HIV-infected individuals, only 40.0% ever tested for HIV. There were no variations across age groups (p =0.15), regions (p = 0.20), marital status (p = 0.07), and perception of professional vulnerability for HIV (p = 0.12) (Table 1). HIV testing was higher in HCWs who reported high-risk sex than HCWs who did not had a high-risk sex (p = 0.001) (Table 1). In multivariable logistic regression analysis (Table 1), male sex, working in laboratory, job duration and high-risk sex were associated with an increased access to HIV testing.

**Table 1:** Univariable and multivariable analysis of having testing for HIV by socio-demographic, sexual behaviour, and HIV infection

	**Total n**	**Users of VCT%**	Univariable OR(95%CI)*	Multivariable** OR(95%CI)
**Gender**				
Men	705	42.6	1.4 (1.1 – 1.7)	1.4 (1.1 – 1.6)
Women	865	34.6	1	1
**Age (years)**				
≤24	115	35.7	1.1 (0.7 – 1.8)	1.1 (0.7 – 1.6)
25-29	338	39.1	1.3 (0.9 – 1.8)	1.2 (0.9 – 1.7)
30-34	370	41.6	1.4 (1.0 – 2.0)	1.4 (1.0 – 2.1)
35-39	264	37.5	1.2 (0.8 – 1.7)	1.2 (0.7 – 1.6)
40-44	223	39.0	1.3 (0.9 – 1.9)	1.2 (0.9 – 1.8)
>44	260	33.1	1	
**Geographical location**				
Rural	467	35.8	1	1
Urban	1103	39.2	1.2 (0.9 – 1.4)	1.2 (1.0 – 1.3)
**Marital status**				
Single	493	41.4	1.2 (0.9 – 1.5)	1.1 (0.8 - 1.4)
Married or living in couple	1077	36.7	1	1
**Job category**				
Nurses /Midwives	1038	39.9	1	1
Students and trainees	258	37.6	0.9(0.7 – 1.2)	0.9(0.8 – 1.2)
Laboratory workers	35	82.9	7.3 (3.0 – 17.7)	7.1 (2.9 – 17.6)
Administrative and manual workers	239	24.7	0.5 (0.4 – 0.7)	0.5 (0.4 – 0.8)
**Years of work experience**				
≤ 1 year	288	31.5	1	1
2 – 10 years	1050	39.3	1.4 (1.1 – 1.9)	1.4 (1.1 – 1.8)
>10 years	230	41.2	1.5 (1.1 – 2.2)	1.5 (1.1 – 2.2)
Professional vulnerability for HIV***				
Not vulnerable to HIV	45	35.6	0.9 (0.5 – 1.6)	
Vulnerable to HIV	1515	38.4	1	
Don’t know	10	10.0	0.2 (0.02 – 1.4)	
High-risk sex****				
Yes	312	46.2	1	1
No	1200	36.3	0.7 (0.5 – 0.9)	0.7 (0.4 -0.9)
**Condom at last sex (with occasional partner)**				
Yes	272	44.1	0.5 (0.3 – 1.0)	0.4 (0.3 – 1.0)
No	40	60.0	1	1
**HCWs HIV-infected**				
Yes	35	40.0	1	1
No	978	38.0	0.9 (0.5 – 1.8)	0.8 (0.5 – 1.7)
No sample	557	38.2	0.9 (0.5 – 1.9)	0.7 (0.6 – 1.7)

* Odds ratio (95% confidence interval).

** Covariates in multivariable logistic regression: gender, location, marital status, age, schools, high-risk sex and HIV infection.

*** HCWs who perceived their occupation vulnerable for HIV.

**** Having a non-cohabitating, non-marital sexual partner in the last 12 months (occasional partner).

### Sexual behaviours

About 21% of HCWs reported having had sex with non regular partners over the last 12 months. Of these, 10.0% reported more than one sexual partner. A sexual encounter with a non regular partner was more frequent among male HCWs than among female HCWs (p < 0.001). In addition, a higher percentage of HCWs in the youngest age group, single HCWs, students and trainees reported having sexual intercourse with non regular partners (Table 2). The proportion of HCWs who reported having had sex with non regular partners tended to be higher among HCWs from rural areas than among HCWs from urban areas (23.4% versus 19.3%, p = 0.07). Results of univariate and multivariate analysis for high-risk sex were unchanged when adjusting analyses for gender, location, marital status, age, job category, professional vulnerability, and HIV status.

**Table 2:** Univariate and multivariate analysis of high-risk sex by socio-demographic and HIV infection

	**Total n**	**High-risk sex§%**	Univariable OR(95%CI)*	Multivariable* OR(95%CI)
**Gender**				
Men	678	34.2	4.9 (3.7 – 6.5)	4.9 (3.6 – 6.4)
Women	834	9.6	1	1
**Age (years)**				
≤29	410	39.5	7.3 (4.5 – 12.1)	7.4 (4.6 – 11.1)
30-34	361	18.6	2.5 (1.5 – 4.3)	2.6 (1.4 – 4.1)
35-39	264	14.8	1.9 (1.1 – 3.4)	1.8 (1.1 – 3.1)
40-44	222	10.4	1.3 (0.7 – 2.4)	1.2 (0.7 – 2.3)
>44	255	8.2	1	1
**Marital status**				
Single	435	48.8	9.3 (7.0 – 12.3)	9.1 (7.0 – 11.0)
Married or living in couple	1077	9.3	1	1
**Geographical location**				
Rural	449	23.4	1.3 (1.0 – 1.7)	1.2 (0.9 – 1.6)
Urban	1063	19.3	1	1
**Job category**				
Nurses/ Midwives	1020	17.3	1	1
Students and trainees	229	43.7	3.7 (2.7 – 5.1)	3.4 (2.6 – 5.0)
Laboratory workers	34	20.6	1.2 (0.5 – 2.8)	1.2 (0.4 – 2.6)
Administrative and manual workers	229	12.7	0.7 (0.4 – 1.1)	0.6 (0.4 – 1.1)
**Years of work experience**				
<1 year	200	22.5	1.5 (0.9 – 2.4)	
2 – 10 years	1162	20.4	1.0 (0.7 – 1.6)	
>10 years	150	20.0	1	
Professional vulnerability for HIV***				
Not vulnerable to HIV	41	24.4	1	
Vulnerable to HIV	1460	26.8	1.1 (0.7 – 2.4)	
Don’t know	11	0.0	-----	
**HCWs HIV-infected**				
Yes	35	11.8	1	1
No	970	22.1	2.2 (0.8 – 7.4)	2.3 (0.7 – 7.1)
No sample	507	18.5	1.8 (0.7 – 6.0)	1.6 (0.6 – 5.4)

§ Having a non-cohabitating, non-marital sexual partner in the last 12 months (occasional partner).

* Odds ratio (95% confidence interval).

** Covariates in multivariable logistic regression: gender, location, marital status, age, schools, and HIV infection.

*** HCWs who perceived their occupation vulnerable for HIV.

Thirteen percent of teachers who had sex with a non regular partner did not use condoms. More female HCWs than male HCWs did not use a condom in their last high-risk intercourse (16.7% versus 7.5%, p = 0.02). Single HCWs were less likely to use a condom at last sex with an occasional partner than married HCWs (22.4% versus 15.7%, p < 0.001). There were no association between condom use and age groups (p = 0.41), job category (p = 0.56), employment duration (p = 0.62) or HIV status (p = 0.22).

## Discussion

Our study is the first to investigate VCT uptake services and sexual behaviour among HCWs in a representative sample in West Africa. The findings revealed a low rate of HIV testing among HCWs in Burkina Faso. Nevertheless, this rate of testing for HIV was slightly higher than that of the general population as reported in the Burkina Faso Demographic and Health Survey in 2003 [18] Zungu Li et al. [19] reported that 91% of HCWs in South Africa had been tested for HIV. Such findings in South Africa are in line with other studies that reported a high level of HIV counselling and testing uptake among healthcare workers [20,21]. However, a study in Zambia among HCWs founded a low level of HIV counselling and testing uptake (33%) [22].

Fear of HIV test results was the major reason stated by participants for not undergoing HIV testing. Similarly, a UK-based study showed that that fear of results and fear of colleagues’ reactions were the main reasons for not undergoing HIV testing [23]. Proximity to a clinic [24], perception of being at risk of HIV infection, [25,26] psychosocial factors such as HIV/AIDS-related stigma and discrimination, [24,25] and concerns about confidentiality [24,25] are possible factors associated with VCT uptake.

There is a high female-to-male difference in the use of HIV testing in Burkina Faso HCWs. Male HCWs were more likely to have been tested for HIV than female HCWs. Data from the demographic and health surveys on prior HIV testing experience, suggest higher testing among females in West African countries, [18,27-29] and in South Africa [30-32]. However, according to the 2005 Ethiopia Demographic Health Survey, 4% of women and 6% of men had ever been tested for HIV [33]. Studies conducted in Zambia, Zimbabwe and the UK reported that acceptance of HIV testing was lower among women than men [20,34,35]. The Zimbabwean study specifically reported that women were allegedly more worried about their HIV status and more fearful of HIV testing than men [34].

Our study also showed that VCT service utilization among HCWs in Burkina Faso was the highest among laboratory workers. Laboratory workers are exposed to occupational injuries, exposing to blood-borne pathogens. In the study among HCWs in South Africa, the majority of HCWs stated that they went for HIV counselling and testing afterwards, mainly to determine their HIV baseline status [19]. In fact, in this study, the group which is less exposed to occupational injury, the administrative and manual workers are less likely have tested to HIV infection.

The results of this study also showed a significant association between undergoing HIV counselling and testing, and participants working experience. Not surprisingly, the more experienced were HCW, the more they had been tested for HIV.

HCWs with sexually risky behaviours were more likely to have used VCT services, as reported by previous studies, [36] suggesting a good self-perception of HIV risk in this group.

Among HIV-infected individuals, more than half never tested for HIV. Studies investigating the outcome of VCT in Africa demonstrated a beneficial impact of VCT in HIV-related sexual risk behaviours [36-39]. In a meta-analysis, the odds of reporting increased number of sexual partners were reduced when comparing participants who received VCT with those who did not [38]. When stratified by serostatus, these results remained significant for those who tested HIV-positive. Additionally, people living with HIV who received VCT exhibited increased odds of using condoms and engaging in protected sex than people living with HIV who did not receive VCT [38]. Moreover, previous studies suggested that HIV counselling and testing of individuals and couples is a cost-effective primary HIV prevention strategy [11,40,41]. In our study, the proportion of high-risk sex tended to be lower among HIV-infected HCWs compared those who were not infected or with no urine sample, but without reaching statistical significance.

Sex with non regular and/or multiple partners was considered as risky sexual behaviours. About one out of five interviewed HCWs reported having had sex with a non regular partner within the 12 months preceding the survey. Regional disparities revealed also that sex with non regular partners was reported mostly in rural areas, a situation that could be attributed to the separation from families and poor social support throughout the year. Younger people are involved in more sexually risky behaviours than adults. Gender, age, and marital status were significantly related to high-risk sex behaviours, which is consistent with previous findings among the general population [18].

The non-response rate was low, which ensures that the sample is representative of the target population. All data in the survey were self-reported. Therefore, some degree of under-reporting of socially unacceptable behaviours and over-reporting of socially desirable behaviours are likely. It should also be noted that this study has been based on data collected in 2003, and since then the absolute level of HIV testing experience among HCWs is likely to have changed substantially along with improved access to HIV testing in the country. However, these data is the first epidemiological survey in West Africa addressing HIV testing and risky behaviours among HCWs.

## Conclusion

HIV testing in HCWs in Burkina Faso appears relatively low with high risky behaviors. It seems influenced by job category, working experience and sexually risky behaviors, along with fear of the result. These findings suggest that ‘HCW-friendly’ VCT centres should be implemented, securing confidentiality among colleagues. In addition, refreshment courses on HIV risk reduction, counselling and testing are certainly required during the professional career of HCWs.

## Abbreviations

HIV/AIDS: Human Immunodeficiency Virus/ Acquired Immune Deficiency Syndrome; VCT: Voluntary counselling and testing for HIV; HRS: High-risk sex.

## Competing interest

The authors have no competing interests to declare.

## Authors’ contributions

The overall study was designed by NN, SY, PF and MCD with input from NM. The field data collection was led by FP and supported by SY and NN. The lab work was done by FKS under the supervision of MCD. FKS carried out all data analyses; FKS and AR interpreted results of statistical testing and made inferences. The manuscript was prepared by FKS, NN, AD, SY, PF, MCD, NM and AR. All authors read and approved the final manuscript.

## Pre-publication history

The pre-publication history for this paper can be accessed here:

http://www.biomedcentral.com/1471-2458/13/540/prepub
